# Recent advances in thin film nanocomposite membranes containing an interlayer (TFNi): fabrication, applications, characterization and perspectives

**DOI:** 10.1039/d2ra06304b

**Published:** 2022-11-29

**Authors:** Jiaqi Wang, Lei Wang, Miaolu He, Xudong Wang, Yongtao Lv, Danxi Huang, Jin Wang, Rui Miao, Lujie Nie, Jiajin Hao, Jianmin Wang

**Affiliations:** Research Institute of Membrane Separation Technology of Shaanxi Province, Key Laboratory of Membrane Separation of Shaanxi Province, Key Laboratory of Northwest Water Resources, Environmental and Ecology, Ministry of Education, Key Laboratory of Environmental Engineering No. 13 Yan Ta Road Shaanxi Province Xi'an 710055 China wl0178@126.com; School of Environmental & Municipal Engineering, Xi'an University of Architecture and Technology No. 13 Yan Ta Road Xi'an 710055 China; Zhongfan International Engineering Design Co. Lian Hu Road, No. 6 Courtyard Xi'an 710082 China

## Abstract

Polyamide (PA) reverse osmosis and nanofiltration membranes have been applied widely for desalination and wastewater reuse in the last 5–10 years. A novel thin-film nanocomposite (TFN) membrane featuring a nanomaterial interlayer (TFNi) has emerged in recent years and attracted the attention of researchers. The novel TFNi membranes are prepared from different nanomaterials and with different loading methods. The choices of intercalated nanomaterials, substrate layers and loading methods are based on the object to be treated. The introduction of nanostructured interlayers improves the formation of the PA separation layer and provides ultrafast water molecule transport channels. In this manner, the TFNi membrane mitigates the trade-off between permeability and selectivity reported for polyamide composite membranes. In addition, TFNi membranes enhance the removal of metal ions and organics and the recovery of organic solvents during nanofiltration and reverse osmosis, which is critical for environmental ecology and industrial applications. This review provides statistics and analyzes the developments in TFNi membranes over the last 5–10 years. The latest research results are reviewed, including the selection of the substrate and interlayer materials, preparation methods, specific application areas and more advanced characterization methods. Mechanistic aspects are analyzed to encourage future research, and potential mechanisms for industrialization are discussed.

## Abstract 2

Polyamide (PA) reverse osmosis and nanofiltration membranes have been applied widely for desalination and wastewater reuse in the last 5–10 years.

## Graphical abstract

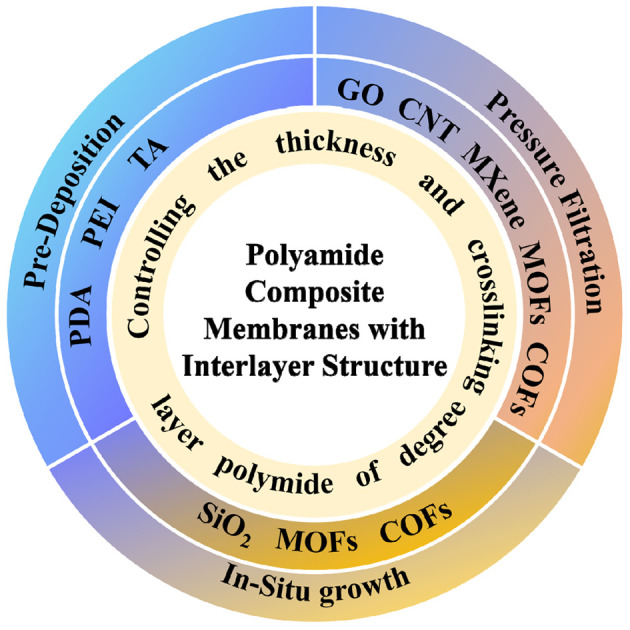

## 1. Introduction

Data indicate that half of the global population is expected to live in water-scarce areas by 2025.^1^ Therefore, water treatment technologies based on membrane separation, such as reverse osmosis and nanofiltration, are playing increasingly important roles in the efficient treatment, recycling and desalination of produced and domestic water.^2–4^ (Reverse osmosis) RO and (Nanofiltration) NF membranes for desalination and recycled water reuse usually have a thin film composite (TFC) structure^4,5^ that consists of two parts: a porous substrate and an ultrathin PA separation layer.^4^ TFC membrane separation layers are usually formed by interfacial polymerization (IP) reactions in aqueous solutions of *m*-phenylenediamine (MPD), *p*-phenylenediamine or piperazine (PIP), which serve as the aqueous phase, and hexane solutions containing trimesoyl chloride (TMC), which serves as the organic phase.

However, the “trade-off” effect between permeate flux and selective separation by TFC membranes has limited their development.^6–8^ In addition, contamination of the PA separation layer on the membrane surface and corrosion by organic solvents have reduced the lifetimes and stabilities of TFC membranes. Researchers have been seeking to develop novel TFC membranes that solve these problems. Therefore, TFN membranes that form nanoscale selective channels utilizing the internal channels of porous nanomaterials and the interfacial channels of PA separation layers have been developed. Conventional TFN membranes are prepared by adding nanomaterials to an MPD aqueous solution or TMC organic phase solution through an IP reaction. Alternatively, a mixed matrix membrane is obtained by adding nanomaterials to the casting solution to modify the substrate.

Different types of nanomaterials, such as SiO_2_,^9^ metal–organic frameworks (MOFs),^10,11^ Ag,^12^ graphene oxide (GO),^13^ and clay,^14^ have been used to prepare TFN membranes. However, the selectivities of many TFN membranes are not substantially increased, and even the retention of the target contaminants is reduced as a result of the irregular distribution or agglomeration of nanomaterials, leading to impaired membrane integrity.

**Fig. 1:**
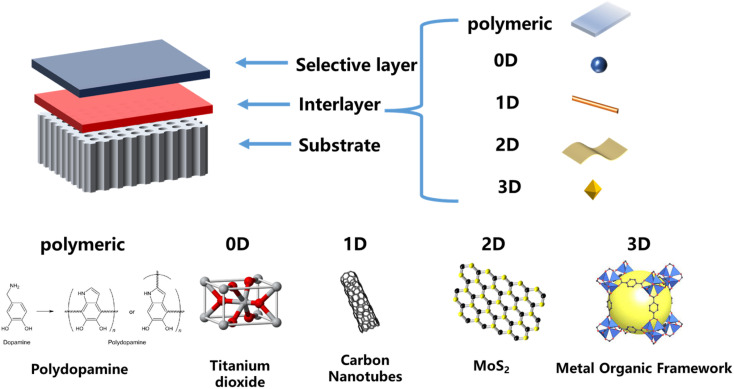
Structure of a TFNi membrane and frequently used intercalation materials.

As shown in Fig. 1, the preparation of TFNi membranes using pressure filtration, predeposition or *in situ* growth of nanomaterials on substrates prior to the formation of PA layers has become a focus of interest. This method consists of 0D (*e.g.*, graphene quantum dots (GQDs),^16^ TiO_2_ (ref. 17) and Ag^18^), 1D (*e.g.*, SWCNTs^19^ and dopamine^20^ and polyethyleneimine^12^), 2D (*e.g.*, MXene, MOFs of the tetrakis(4-carboxyphenyl)porphyrin (TCPP) series and covalent organic frameworks (COFs) of 2D TpPa, *etc.*) and 3D (*e.g.*, UiO-66 and ZIF-8,^21^*etc.* MOFs) nanomaterials in combination. Surface coating,^20^ predeposition,^22^*in situ* growth^23^ and sacrificial intercalation nanomaterials^17^ are used to prepare TFNi membranes.

**Fig. 2:**
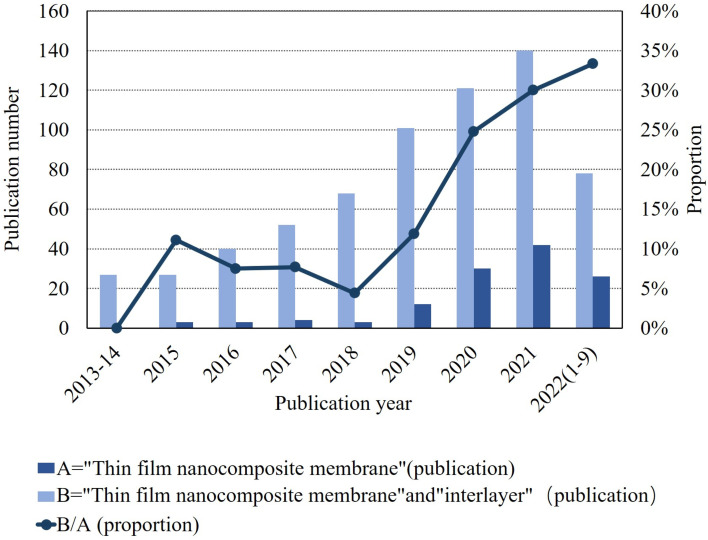
Recent articles describing TFN and TFNi membranes published in the last decade. Data for 2022 are incomplete. All data were obtained by searching the Web of Science database with the keywords “thin film nanocomposite membrane” and “interlayer” and calculating their ratios.

As shown in Fig. 2, a 2022 literature search using the Web of Science search system with interlayers as the subject keyword identified approximately 35% of all TFN membranes, and the trend has been increasing annually over the past 10 years. To date, several review articles related to the preparation of polyamide composite membranes through intercalation have been published. Among them, Liao *et al.*^24^ targeted the selection of different types of nanofillers based on TFN membranes in application scenarios such as gas separation, nanofiltration, organic solvent nanofiltration, reverse osmosis, forward osmosis, and osmotic evaporation. Seah *et al.*^25^ and Bhaskar, V. V. *et al.*^26^ reviewed the IP processes of TFN and TFC membranes, including support-free IP, filtration-based IP, spin-based IP, ultrasound-based IP, and spray-based IP. The intent was to compare which IP process is more likely to be used in real production and is environmentally friendly. Zhao *et al.*^27^ reviewed the application of TFN membranes in RO with the intention of investigating the effect of nanofillers on the polyamide layer and the improvement of membrane contamination resistance in practical water treatment processes. Zhang *et al.*^28^ reviewed organic nanomaterial-modified TFN membranes and their research progress in various water/organic separation processes. The intention was to explore the possible applications of organic nanomaterial-modified TFN membranes in practical water treatment projects. Yang *et al.*^29^ intended to investigate the advantages of TFNi membranes compared to TFN and TFC membranes through basic theory and to determine the effect of different nanomaterials on the polyamide layer.

Compared to the aforementioned reviews, this paper mainly focuses on the preparation methods and categorization of TFNi membranes with the intention of exploring the possibility of scaling up their production toward practical water treatment projects and summarizes several conventional techniques for the characterization of TFNi membranes.

## 2. Properties of different substrate materials

TFNi membranes usually consist of a substrate, a PA layer and a nanomaterial sandwich. The physicochemical properties of the substrate directly influence the selection of the interlayer nanomaterial and the properties of the PA layer. Therefore, a suitable substrate should be selected according to the different physicochemical properties of the target contamination.

Polyimide (PI) membranes have been used in membrane technology for many years and have a wide range of applications. PI membranes are excellent polymers for the preparation of substrates because of their excellent heat resistance and good mechanical strength, as well as chemical resistance to a variety of solvents.^30^ PI membranes were initially used for gas separation^31^ and permeate evaporation^32^ due to their high mechanical strengths. In addition, due to their high organic solvent resistance, they have become an important part of organic solvent nanofiltration processes, and these PI membranes are currently used to recover various organic solvents.^33–36^

Polysulfone (PSf) has good chemical resistance to alkaline solutions, acids, and chlorine, and it has high mechanical strength and is easily processed. Therefore, PSf is used as a raw material for the preparation of microfiltration and ultrafiltration membranes, as well as a substrate material for the preparation of reverse osmosis and nanofiltration membranes.^37^ However, the hydrophobicity of PSf tends to make the membrane surface susceptible to contamination.^38,39^ Therefore, the surface of the membrane is modified to enhance its resistance to contaminants by increasing its hydrophilicity to improve its long-term stability in water treatment processes.^40^

Polyethersulfone (PES) is a typical industrial water treatment membrane. One of its major applications is the removal of various organic and inorganic heavy metals and dyes from wastewater.^41^ It has excellent mechanical properties and heat resistance.^42^ Therefore, membranes synthesized from PES can be fabricated into structures suitable for various wastewater treatment methods. Despite the remarkable excellence of PES as a substrate material, it is, like PSf, susceptible to contamination, which reduces its treatment efficiency.^43^

Polyvinylidene fluoride (PVDF) has received increasing attention as a membrane material with a high mechanical strength, thermal stability and corrosion resistance comparable to those of other commercial polymer materials. PVDF membranes have been widely used in membrane separation applications such as ultrafiltration and microfiltration.^44^ However, PVDF is less commonly used for the recovery of organic solvents because it is readily soluble in organic solvents such as ethanol and *N*,*N*-dimethylformamide (DMF).^45^

In addition to these polymers, others, such as Nylon, PSU,^46^ PPS,^47^ SPEEK^12^ and CTA,^48^ have been used as TFNi membrane substrates for water treatment. Notably, each substrate has its own unique advantages and characteristics and should be selected appropriately according to the practical operating environment. In the context of the *in situ* growth method, a substrate is needed as a support during synthesis. Therefore, the mechanical strength, acid resistance and temperature range to which the substrate are subjected during the growth process are considered. All these factors limit the selection of substrates and nanomaterials.

## 3. Selection of interlayer materials

The physicochemical properties of the intercalated material can be used to control the diffusion concentrations of amine monomers in the aqueous phase during the IP reaction. In addition, if the intercalated material is a two- or three-dimensional nanomaterial, the target material can be processed using its own formed Å-sized layer spacings or microwindows. Therefore, the choice of interlayer nanomaterial exerts a substantial effect on the performance of a TFNi membrane.

Zero-dimensional nanomaterials, such as graphene quantum dots (GQDs), constitute a new class of graphene oxide materials consisting of single-atom (<2 nm) nanosheet structures with high specific surface areas and excellent chemical stabilities. Deposition of an appropriate amount of GQDs on the surface of the substrate may subsequently reduce the thickness of the PA layer, which helps to improve the permeabilities of TFN membranes.^15,16^

Tannic acid (TA), a plant processing product, easily modifies the surfaces of substrates *via* complexation reactions with metal ions and organic substances because of its abundant hydroxyl groups and hydrogen bonds. For example, complexes formed from tannic acid (TA) and Cu^2+^,^49^ Fe^3+^,^50^ polyethyleneimine (PEI),^51^ polydopamine (PDA),^20^ and diethylenetriamine (DETA)^52^ were used as intercalation nanomaterials to modify TFNi membranes.

Polyethyleneimine (PEI), a cationic polyelectrolyte with a high charge density and easy protonation, has been widely used to prepare positively charged TFC membranes. Polyphenols composed of PEI and TA complexes have been deposited on the surface of the substrate, and the formation of a PA separation layer on the surface of the substrate was analyzed by determining the diffusion kinetics of fluorescence-labeled piperazine (FITC-PIP), the diffusion behavior of an *n*-hexane solution containing chlorinated chloride, and with *in situ* Fourier transform infrared spectroscopic (FT-IR) studies.^51^ PEI deposition of a carboxylated polyacrylonitrile (PAN) substrate was used to control the subsequent IP reaction by modulating the diffusion rate of the –NH_2_ monomer with PEI.^53,54^ In addition, the hydrophilic interlayer formed by PEI and PDA codeposition also controls the subsequent IP reaction.^47,48,55,56^

Dopamine self-polymerizes into PDA under weakly alkaline conditions at pH = 8, and the surface of the substrate forms a stable surface coating. PDA provides a versatile platform for the subsequent modification of nanomaterials with physical and chemical interactions.^57^ Using PDA as a representative adhesive material, the mechanism of action for intercalation materials deposited on TFNi membranes can be explored.^58^

MOFs, also known as porous coordination polymers, are widely used in adsorption and membrane separations due to their adjustable porosities, dimensionalities, and internal pore channels.^59^ Various methods have been proposed to improve interfacial compatibility issues with MOFs and substrates. These methods include an *in situ* growth strategy, in which the zeolite-imidazolite framework (ZIF-8) and the *in situ* growth of ZIF-8 form a uniformly distributed intercalation material on the surface of the substrate.^60^ Similar to this method, PI was used as a substrate, and PI was preimmersed in a Cu^2+^ solution; subsequently, HKUST-1 nanomaterials were grown *in situ* on the PI surface using a low-temperature solvothermal method.^61^ The photocatalytic properties of specific MOF nanomaterials have improved the anti-pollution properties of membranes. If copper-triazole^62^ or Fe-TCPP^63^ was pressure filter loaded onto the substrate surface, the resulting TFNi membranes were self-cleaned by applying light during the subsequent dye wastewater treatment, which improved the water treatment stability and enabled good flux recovery.

COFs are emerging as porous crystals. COFs are two- or three-dimensional polymers connected by covalent bonds, and they have unique properties, such as tunable pore channels, high specific surface areas, and chemical stability.^64,65^ TpPa nanomaterials are often used as COFs in membrane separations because they are synthesized at ambient temperature and pressure, grown *in situ* and loaded on the surface of the substrate without damaging the membrane structure.^66–71^

GO is a frequently applied two-dimensional material that has been used in TFNi membranes, but GO is less stable in aqueous solutions.^72^ GO is usually modified with hydrophilic polymers such as PDA and PEI to solve this problem.^12,47,73^ Alternatively, carbon nanotubes (CNTs) are blended with GO^74^ and pressure filtered onto the surface of the substrate to serve as an intercalation material. In addition, two-dimensional MXene materials are more frequently used as interlayer-modified TFNi membranes due to the large number of hydroxyl hydrophilic functional groups on the surface. However, the disadvantage of MXenes is that they will be oxidized to TiO_2_ when exposed to air for a long time,^75^ and this property can be used to prepare sandwich material-free modified TFC membranes by flushing the sandwich material TiO_2_ with water.^17^

Different preparation methods correspond to the selection of different intercalation materials, and the preparation methods are divided into three categories: predeposition, pressure filtration and *in situ* growth. The strengths and weaknesses of these three loading methods and the materials used with each preparation method are indicated and summarized in each subsequent section.

## 4. Different methods for preparing TFNi membranes

Three categories are summarized here, depending on the loading method of the interlayer material, namely, predeposition, pressure filter loading and *in situ* growth. Each preparation method corresponds to the specific properties of interlayer nanomaterials, and the researcher should select the appropriate interlayer material according to the actual application scenario (*e.g.*, NF, RO, or FO). (1) The pressure filtration method has recently attracted increasing attention from researchers due to its convenient and controlled preparation process and low material consumption. The preparation process uses one-dimensional or two-dimensional materials as interlayer materials, and the interlayer materials are uniformly distributed on the substrate surface in a regular shape by applying an external force. The physical and chemical properties of the intercalated material are mainly used to control the subsequent formation of the PA layer on its surface. (2) The representative material of the TFNi membrane prepared using the predeposition method is a polymer, which regulates the subsequent formation of the PA layer on the substrate surface through the adhesion of the polymer. (3) *In situ* growth typically uses the substrate as a support and occurs directly on the substrate surface during the synthesis of the material. The applicable water treatment scenarios for each of the three preparation methods and the respective representative materials are discussed in detail in the subsequent sections.

### 4.1 Pressure filtration

The pressure filtration method is performed by applying pressure to filter the nanomaterial dispersion onto the substrate surface, followed by an IP reaction on its surface to obtain the TFNi membrane. The preparation method may be inspired by laminated membranes of 2D materials because the process used to prepare TFNi membranes with the pressure filter loading method is similar to that of laminated membranes, except for the IP reaction, as the nanofolds are similar to those formed by laminated membranes composed of 2D materials.

Due to limitations on the thickness of the interlayer material in the preparation of TFNi membranes using the pressure filter loading method, an excessively thick interlayer may decrease the stability of the PA separation layer, and thus a high separation performance is impossible to maintain for a long time. Therefore, the thickness of the sandwich material of a TFNi membrane is usually approximately 5–10 nm.

Furthermore, water molecule transport through the channels of laminated membranes involves interlayer transport, and the resulting wrinkles help to accelerate transport to a certain extent. Similarly, TFNi membranes have been constructed by intercalating materials to create a selective layer with folds that accelerate water transport. For example, Guo *et al.* prepared TFNi membranes by pressure filter loading sulfonated polyaniline SPANI nanocellulose as an intercalation material. After grafting sulfonic acid groups on polyaniline, the electronegativity of the whole membrane was significantly increased, which increased the repulsive force for divalent ions.^76^ Excellent water permeability and selectivity for mono- and divalent ions were observed. Second, SPANI nanofiber interlayers provide hydrophilic porous surfaces that contribute to the formation of defect-free and thin PA selective layers with pleated nanostructures. Doping ions into the interior of the polymer forms stable six-membered rings in the self-doped structures, decreasing the susceptibility of the polymer to damage by weakly acidic and weakly basic solutions. The stability of the TFNi membrane is improved.^77^

**Fig. 3:**
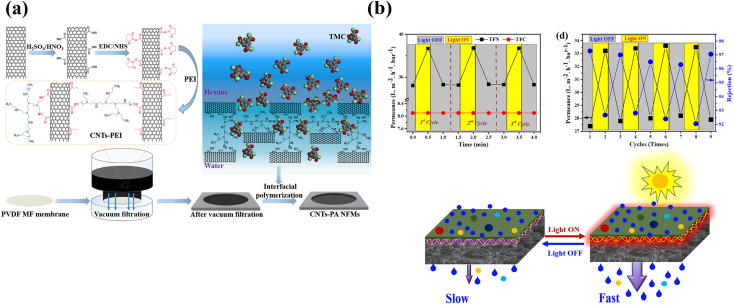
(a) PEI was grafted onto the surface of CNTs and deposited on a PVDF substrate. The grafting of PEI resulted in an amine-rich interlayer [reprinted with permission from ref. 79. Copyright 2021, Elsevier]. (b) Relative pure water permeance of TFC and TFN membranes as a function of the illumination time, effect of light illumination for 0.5 min on pure water permeance and schematic illustrating the photothermal effect on the TFN NF membrane after lights were switched ON and OFF [reprinted with permission from ref. 63. Copyright 2022, Elsevier].

Removal of target contaminants has been enhanced by changing the charge of the intercalated material. For example, Li *et al.* mixed positively charged PEI and negatively charged MXene nanosheets onto the surface of a PAN substrate for filtration and applied the obtained TFNi membrane to dye retention.^78^ As shown in Fig. 3(a), Chen *et al.* used PEI mixed with CNTs and loaded it onto the surface of a PVDF substrate by filtration. CNT TFNi membranes were obtained and applied to dye retention, and the retention performance was adjusted by changing the pH (2.12% pH = 12, 99.38% pH = 7).^79^

The “ridge and valley” feature on the surface of the PA layer is caused by the release of nanobubbles encapsulated in the PA layer during the IP process.^80,81^ This phenomenon may be attributed to two factors: (1) the heat generated reduces the solubility of dissolved gases in the aqueous solution because the IP process requires a thermal cross-linking reaction; and (2) the protons generated during the IP process form H_2_CO_3_ from HCO^3−^, which accelerates the formation of CO_2_ in the thermal cross-linking environment at 60 °C. Sun *et al.* prepared MXene/CNTs TFNi membranes to increase the adsorption of amine monomers in the aqueous phase solution onto the intercalated layer and to limit bubble generation during IP reactions.^82^ Similar to the process shown in Fig. 3(b), Wen *et al.* prepared two-dimensional MOF (Zn-TCPP) TFNi membranes to enhance the confinement of degassing nanobubbles, which reduced the diffusion rate of –NH_2_ monomers.^83^ In addition, two-dimensional MOFs of the porphyrin family constitute a class of photothermal sensitive devices^84^ that were used as tunable photoresponsive TFNi membranes by Hussain *et al.* The ultrafast photothermal responses of Fe-TCPP (2 s of light irradiation increased the temperature by 88.4 °C) resulted in a 30% increase in water flux through the membrane during visible light irradiation for 30 s.^63^

**Fig. 4:**
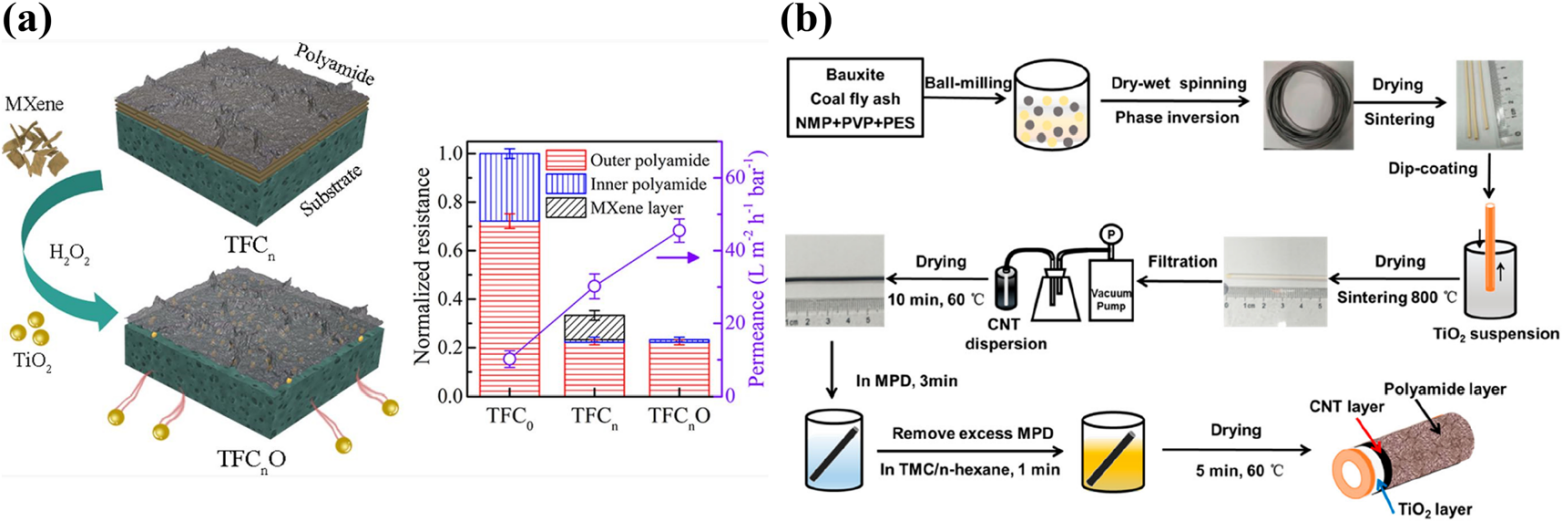
(a) The MXene lamellar layer was eliminated by mild oxidation after IP to avoid imparting additional hydraulic resistance [reprinted with permission from ref. 17. Copyright 2021, American Chemical Society]. (b) Schematic diagram of the preparation process for a ceramic-based FO membrane with a TiO_2_/CNT nanocomposite interlayer [reprinted with permission from ref. 87. Copyright 2020, American Chemical Society].

Although the leaching of nanomaterials from TFNi membranes prepared using pressure filtration has been substantially reduced during the treatment process compared to predeposition and *in situ* growth methods, the leaching of nanomaterials is still a concern that requires a solution. As shown in Fig. 4(a), Xu *et al.* converted the PES substrate after filtering MXene into TiO_2_ using mild oxidation of H_2_O_2_ and flushed it away after the IP reaction to form a PA layer as an approach to solve this problem. The resulting TFC membrane conferred a high rejection rate >96% for Na_2_SO_4_ and an excellent permeance of 45.7 L·m^−2^ h^−1^ bar^−1^, which was approximately 4.5 times higher than that of the control membrane (10.2 L m^−2^ h^−1^ bar^−1^). This method fully uses the physicochemical properties of the MXene nanomaterial, eliminates the additional hydraulic resistance defects of the intercalated material, and solves the negative effect on environmental water after leaching.^17^

Most of the intercalation materials that can be used in pressure filtration preparation processes are nanomaterials with one- or two-dimensional morphology. Zhu *et al.* used cellulose nanocrystals (CNCs) and GO, both alone and combined, as intercalation materials to investigate the performance of TFNi membranes. The experimental results showed an effective reduction in the surface roughness of the interlayer and syntheses of 10–15 nm ultrathin PA layers with a low nanomaterial concentration of 0.025 wt%.^85^

Tian *et al.* filtered GO onto the outer surface of a PSf hollow fiber (HF) substrate to prepare HF nanofiltration membranes, and the surface morphology and cross-sectional structure of the prepared TFNi nanofiltration membrane were investigated extensively.^86^ As shown in Fig. 4(b), Jin *et al.* obtained mechanically strong TFNi membranes by growing TiO_2_ on the surface of a PES HF substrate and subsequently loading CNTs on the TiO_2_ surface *via* filtration. The structural morphologies and properties of the nanomaterial-free interlayers, TiO_2_ interlayers and TiO_2_/CNT interlayered TFNi membranes were characterized. The introduction of nanocomposite interlayers with lower roughness resulted in TFNi membranes with better surface properties and facilitated the formation of defect-free PA separation layers with higher cross-linking.^87^

Although membrane separation technology has great potential in dye wastewater treatment, a common problem is substantial decreases in separation effectiveness and lifetime due to membrane contamination. Zhou *et al.* exploited the photocatalytic properties of the copper triazolate (CuTz-1) MOF to degrade organic molecules with visible light.^88^ The MOFs (CuTz-1) and GO were filtered and chemically cross-linked to construct (CuTz-1)/GO intercalated TFNi membranes. The presence of CuTz-1 led to an increase in the GO interlayer distance, thus promoting enhanced water flux without affecting dye retention. Moreover, after visible light irradiation, the permeation and separation properties of the TFNi membrane recovered well due to effective photocatalytic removal of the dye attached to the membrane surface.^62^

Compared to the internal and interfacial channels of typical TFN membranes, which contain channels measuring in the subnanometer to low nanometer range, the nanochannels generated by sacrificial nanomaterials usually have larger sizes (>10 nm). For example, Yang *et al.* incorporated sacrificial Cu NPs into a PA rejection layer.^89^ The nanocavities created by subsequent acid etching resulted in a 3-fold increase in water flux at the cost of a slight reduction in NaCl retention.

The aforementioned representative cases of TFNi membranes prepared using the pressure filtration method are selected from Table 1 according to their preparation methods and target contaminants. Table 1 also provides a summary of TFNi membranes prepared using the pressure filtration method.

**Table 1:** Summary of pressure filtration methods for preparing TFNi membranes

Substrate material	Interlayer nanomaterials	Membrane type	Operating conditions (applied pressure and feed solution)	Permeability (L m^−2^ h^−1^ bar^−1^)	Rejection (%)	Reference
PAN	PEI/MXene	NF	Na_2_SO_4_ and 1000 mg L^−1^, dyes 200 mg L^−1^	20.9 L m^−2^ h^−1^ bar^−1^	*R*_Na_2_SO_4__ = 65.7%, *R*_NaCl_ = 23.9%, *R*_Congored_ = 99.42%, *R*_reactiveblue_ = 99.02%, *R*_methylblue_ = 98.84%	78
PES	β-Cyclodextrin (β-CD)/GO	NF	Crystal violet 10 ppm, Congo red 100 ppm, methylene blue 10 ppm, Na_2_SO_4_, NaCl and MgCl_2_ 1000 ppm	82 L m^−2^ h^−1^ bar^−1^	*R*_crystalviolet_ = 99.98%	92
PVDF	CNTs-PEI	NF	Na_2_SO_4_ and NaCl 1000 ppm, Congo red 100 ppm	49.8 L m^−2^ h^−1^ bar^−1^	*R*_Congored_ = 99.47%, separation α (NaCl/CR) = 150.32	79
PSf	Hydroxyapatite (HAP)	NF	Na_2_SO_4_ and NaCl 1000 ppm	177 L m^−2^ h^−1^ 6 bar	*R*_Na_2_SO_4__ = 98.2%, separation α (NaCl/Na_2_SO_4_) = 19	94
PAN	CuTz-1	Self-cleaning NF	Na_2_SO_4_ and NaCl 1000 ppm, crystal violet 500 ppm, Congo red 500 ppm, methylene blue 500 ppm	40.2 L m^−2^ h^−1^ bar	*R*_Na_2_SO_4__ = 19.6%, *R*_NaCl_ = 0.3%, *R*_Congored_ = 99.4%, *R*_directred_ = 98.2%, *R*_methylblue_ = 94.9%	62
PES	CNCs/PDA	NF	Na_2_SO_4_ and NaCl sodium, lignosulfonate, alkaline lignin, and BSA 500 ppm, Congo red 500 ppm, rhodamine B 500 ppm	19.0 L m^−2^ h^−1^ bar	*R*_Na_2_SO_4__ = 98.2%	95
PSf	GO	NF	Na_2_SO_4_ 2000 ppm	80 L m^−2^ h^−1^ at 0.6 MPa	*R*_Na_2_SO_4__ = 96.1%	86
PSf	CNCs/GO	NF	Na_2_SO_4_ 1000 ppm	45.9 L m^−2^ h^−1^ bar	*R*_Na_2_SO_4__ = 97.2%	85
PSf	g-C_3_N_4_/halloysite nanotubes (HNTs)/PIP	NF	Na_2_SO_4_ 1000 ppm	20.5 L m^−2^ h^−1^ bar	*R*_Na_2_SO_4__ = 94.5%	96
PES	Carboxylated CNCs (CNC–COOH)	NF	LiCl and MgCl_2_ 2000 ppm	4.17 L m^−2^ h^−1^ bar	Separation α (Mg^2+^/Li^+^) = 12.5	97
PES	CNS	NF	Na_2_SO_4_ 1000 ppm	204 g m^−2^ h^−1^ at 0.6 MPa	*R*_Na_2_SO_4__ = 97%	98
PES	MXene/TiO_2_	NF	Na_2_SO_4_ 1000 ppm	45.7 L m^−2^ h^−1^ bar	*R*_Na_2_SO_4__ = 96%	17
PES	Fe-TCPP	NF	Methyl orange, indocyanine green, brilliant blue and direct red 30 ppm	28.1 L m^−2^ h^−1^ bar	*R*_methylorange_ = 84.5%, *R*_indocyaninegreen_ = 97.2%, *R*_brilliantblue_ = 98.3%, *R*_directred_ = 99.1%	63
PPS	NGO	NF	NaCl, CuSO_4_, ZnSO_4_ and AlCl_3_ 1000 ppm	40.80 L m^−2^ h^−1^ at 0.6 MPa	*R*_NaCl_ = 48.24% *R*_CuSO_4__ = 92.14%, *R*_ZnSO_4__ = 92.93% *R*_AlCl_3__ = 91.23%	47
Polytetrafluoroethylene (PTFE)	PDA/SWCNT	OSN	Congo red 20 ppm	13.2 L m^−2^ h^−1^ bar	*R*_HNSA_ = 89.6%	99
PES	PDA/NMG/SiO_2_	OSN	Acid red 18	2.18 L m^−2^ h^−1^ bar	*R*_acidred18_ = 99.9%	100
PAN	Ethylenediamine modified graphene oxide (eGO)	OSN	Water/isopropanol separation	4150 g m^−2^ h^−1^	Separation α (water/isopropanol) = 1866	101
PES	Zn-TCPP	RO	NaCl 2000 ppm	4.82 L m^−2^ h^−1^ bar	*R*_NaCl_ = 97.4%	83
PSf	PEI/GO	RO	NaCl 2000 ppm	2.24 L m^−2^ h^−1^ bar^−1^	*R*_NaCl_ = 96.6%	91
PES	MXene/CNT/PDA	FO	0.5, 1, 2 and 3 mol per L NaCl	31 L m^−2^ h^−1^	Js NaCl 0.07 g m^−2^ h^−1^	82
PES	GO quantum dots (GOQDs)/Ag	FO	1 mol per L NaCl	65.8 L m^−2^ h^−1^	Js NaCl 1.4 g m^−2^ h^−1^	102
PES	GO	FO	2 mol per L NaCl	22 L m^−2^ h^−1^	Js NaCl 5.8 g m^−2^ h^−1^	93
PAN	PDA	FO	2 mol per L NaCl, Cr^3+^, Cu^2+^ and Ni^2+^ 20 mg L^−1^	29.2 L m^−2^ h^−1^	*R*_Cr^3+^_ = 99.5%, *R*_Cu^2+^_ = 99.1%, *R*_Ni^2+^_ = 98.1%	103
PVDF	GO/CNTs	FO	0.6 mol per L NaCl	305.89 L m^−2^ h^−1^	Js NaCl 0.37 g m^−2^ h^−1^	74
PES	TiO_2_/CNT	FO	0.5, 1 and 2 mol per L NaCl	2.0 L m^−2^ h^−1^ bar	*R*_NaCl_ = 97.2%	87
Nylon	MXene	FO	5 mol per L monoethanolamine (MEA) solution	30.8 L m^−2^ h^−1^ bar	CO_2_ capture	90
PES	CNT	FO	1 mol per L NaCl	35 L m^−2^ h^−1^ bar	Js NaCl 0.55 g m^−2^ h^−1^	104

The intercalation materials used in the pressure filtration method are mostly 1D or 2D materials or their modified composites, with representative 2D materials including MXenes,^17,78,82,90^ metal-TCPP^63,83^ and GO.^47,74,85,86,91–93^ This phenomenon may be attributed to the three factors listed below. (1) The pressure filtration method enables orderly loading onto the substrate surface, which maximizes the advantages of the physicochemical properties of 2D materials, as well as the Å level layer spacing. (2) The exfoliated monolayer 2D materials are generally uniformly dispersed in deionized water or organic solvents. Therefore, if the TFNi membranes composed of 2D materials are prepared using pressure filtration, the experimental reproducibility is high, and other experimental conditions can be easily investigated. (3) Compared to predeposition and *in situ* growth, pressure filtration exhibits close to one hundred percent utilization of the material. Therefore, the preparation process is also the least polluting to the environment.

Currently, due to its limited preparation device requirements, most of the TFNi membranes prepared using the pressure filtration method have an area of approximately 10–100 cm^2^, and thus further industrialization is challenging. Second, although the material utilization rate of the pressure filtration method is relatively efficient, the cost of currently used materials remains expensive if we want to further expand production. Moreover, the environmental pollution caused by the secondary leaching of the intercalated material in the treatment process is also a problem that we must consider.

### 4.2 Predeposition

The predeposition method usually consists of covering the material on the substrate surface by deposition using its physicochemical properties to control the diffusion rate of the amine monomer to the organic phase, thereby regulating the subsequent IP reaction. Compared to Table 1, which summarizes the pressure filtration method for preparing TFNi membranes, as shown in Table 2, the predeposition method uses a wide range of polymers, such as PDA,^58^ PIPA,^105^ PEI^56,106^ and SA (sodium alginate),^107^ as additional intercalation materials. Using this approach, the cost of the preparation process and the secondary pollution to the environment in water treatment are substantially reduced. Second, it provides the possibility to further expand the production of TFNi membranes.

**Table 2:** Summary of predeposition methods for preparing TFNi membranes

Substrate material	Interlayer nanomaterials	Membrane type	Operating conditions (applied pressure and feed solution)	Permeability (L m^−2^ h^−1^ bar^−1^)	Rejection (%)	Reference
PES	3-Aminobenzenesulfonamide (ABSA)	NF	NaCl, MgSO_4_, Na_2_SO_4_ and MgCl_2_ 1000 ppm, dye solutions 0.1 g L^−1^	12.4 L m^−2^ h^−1^ bar^−1^	*R*_Na_2_SO_4__ = 95%, *R*_dyes_ = 99.3%	120
PES	GO/PDA	NF	MgSO_4_, NaCl, Na_2_SO_4_ and MgCl_2_ 1000 ppm	11.4 L m^−2^ h^−1^ bar^−1^	*R*_NaCl_ = 66.99% *R*_MgSO_4__ = 97.76%, *R*_MgCl_2__ = 84.05% *R*_Na_2_SO_4__ = 98.22%	73
PSf hollow fiber	TpPa (COFs)	NF	ZnSO_4_, CuSO_4_, Cr_2_(SO_4_)_3_, Na_2_SO_4_ and MnSO_4_ 2000 ppm	86.6 L m^−2^ h^−1^ Mbar^−1^	*R*_ZnSO_4__ = 91.7%, *R*_MnSO_4__ = 91.0%, *R*_Cr_2_(SO_4_)_3__ = 95.4% *R*_Na_2_SO_4__ = 96.6%, *R*_CuSO_4__ = 94.3%	68
PES	MXenes	NF	NaCl and Na_2_SO_4_ 1000 ppm	27.8 L m^−2^ h^−1^ bar^−1^	Separation α (NaCl/Na_2_SO_4_) = 480	121
PSf	PEI-Noria	NF	MgSO_4_, NaCl, Na_2_SO_4_ and MgCl_2_ 2000 ppm	110.4 L m^−2^ h^−1^ Mbar^−1^	*R*_MgCl_2__ = 95.4%	106
PSf	Catechol/sodium periodate	NF	Na_2_SO_4_, NaCl, CaCl_2_ NaCl and MgCl_2_ 2000 ppm	15.4 L m^−2^ h^−1^ bar^−1^	*R*_Na_2_SO_4__ = 98.42%	122
PAN	TpPa (COFs)	NF	MgSO_4_, NaCl 2000 ppm, acid fuchsia, Congo red 100 ppm	104.8 L m^−2^ h^−1^ (0.6 MPa)	Separation α (NaCl/acid fuchsia = 402.5, NaCl/Congo red = 398.0)	69
PSf	PVA/GO	NF	Na_2_SO_4_ and NaCl 2000 ppm	158 L m^−2^ h^−1^ MPa^−1^	*R*_Na_2_SO_4__ = 99.7% separation α (NaCl/Na_2_SO_4_) = 230	116
PSf	Homogeneous gelatin (GT)	NF	Na_2_SO_4_, CaCl_2_, NaCl and MgSO_4_ 2000 ppm	16.95 L m^−2^ h^−1^ bar^−1^	*R*_Na_2_SO_4__ = 99.3% separation α (NaCl/Na_2_SO_4_) = 97.73	123
PES	ZIF-8	NF	Na_2_SO_4_, NaCl 500 ppm, acid fuchsia, Congo red 100 ppm	9.6 L m^−2^ h^−1^ bar^−1^	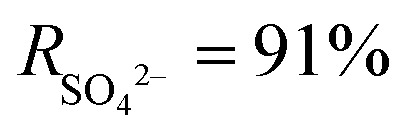	124
PES	SWCNT	NF	Na_2_SO_4_, NaCl, MgSO_4_ and MgCl_2_ 1000 ppm	18.24 L m^−2^ h^−1^ bar^−1^	*R*_Na_2_SO_4__ = 97.88%	19
PES	Iron oxohydroxide (FeOOH) nanorods	NF	Na_2_SO_4_, NaCl, MgSO_4_ and MgCl_2_ 2000 ppm	34.1 L m^−2^ h^−1^ bar^−1^	*R*_Na_2_SO_4__ = 95.0% separation α (Cl^−^/SO_4_^2−^) = 97.73	125
PSf	PDA	NF	Na_2_SO_4_, MgSO_4_ and MgCl_2_ 1000 ppm	14.8 L m^−2^ h^−1^ bar^−1^	Separation α (Cl^−^/SO_4_^2−^) = 34.4	58
PES	PVA	NF	Na_2_SO_4_ 2000 ppm	31.4 L m^−2^ h^−1^ bar^−1^	*R*_Na_2_SO_4__ = 99.4%	115
PES	TpPa (COFs)	NF	Na_2_SO_4_ 1000 ppm	171.35 L m^−2^ h^−1^ MPa^−1^	*R*_Na_2_SO_4__ = 90%	126
Polyphenylene	PDA/PEI/GO	NF	AlCl_3_, NaCl, ZnSO_4_ and CuSO_4_ 1000 ppm	40.8 L m^−2^ h^−1^ MPa^−1^	*R*_ZnSO_4__ = 92.93%, *R*_AlCl_3__ = 91.23%, *R*_NaCl_ = 48.24%, *R*_CuSO_4__ = 92.14%	47
Ceramic	Poly(4-styrenesulfonic acid) (PSS)/ZIF-8	NF	Water–ethanol	4.47 kg m^−2^ h^−1^	*P*_w_/*P*_e_ = 318–127	127
PAN	PDA/COF	NF	Orange GII 100 mg L^−1^, Na_2_SO_4_ 1000 mg L^−1^	207.07 L m^−2^ h^−1^ MPa^−1^	*R*_Na_2_SO_4__ = 93.4%, *R*_orangeGII_ = 94.5%	71
PES	CNT	NF	Methyl violet 100 mg L^−1^, Na_2_SO_4_, NaCl, MgSO_4_ 2000 ppm	21 L m^−2^ h^−1^ bar^−1^	*R*_Na_2_SO_4__ = 98.3%, *R*_MgSO_4__ = 98.3%, *R*_methylviolet_ = 99.5%, separation α (Cl^−^/SO_4_^2−^) = 85.5, (NaCl/methyl violet) = 123.5	128
PSf	PEI/PDA	NF	Na_2_SO_4_ and MgSO_4_ 1000 ppm	45 L m^−2^ h^−1^ at 0.6 MPa	*R*_Na_2_SO_4__ = 97%	56
PSf	TA/DETA	NF	Na_2_SO_4_ and MgSO_4_ 2000 ppm	63 L m^−2^ h^−1^ at 0.6 MPa	*R*_Na_2_SO_4__ = 98%	52
Polyimide (PI)	TpPa (COFs)	OSN	Rhodamine B 100 mg L^−1^	60 L m^−2^ h^−1^ Mbar^−1^	*R*_Rhodamineb_ = 99.2%	67
Polyketone (PK)	PDA/GQD_NH2_	OSN	Rhodamine B 20 ppm	11.1 L m^−2^ h^−1^ bar^−1^	*R*_Rhodamineb_ = 99%	15
Polyamide 6 hollow fiber	Silicate sheets	OSN	Vitamin B_12_ 100 mg L^−1^	0.1 L m^−2^ h^−1^ bar^−1^ (MeOH)	*R*_VB_ = 99%	129
PI	ZIF-8	OSN	Rhodamine B	28 L m^−2^ h^−1^ MPa^−1^ (ethanol)	*R*_Rhodamineb_ = 99.1%	130
PI	TA–Cu	OSN	Rhodamine B 100 mg L^−1^	150.05 L m^−2^ h^−1^ MPa^−1^	*R*_Rhodamineb_ = 97.8%	49
Polyimide polymer (P48)	GQDs/PEI	OSN	Rhodamine B	40.3 L m^−2^ h^−1^ MPa^−1^	*R*_Rhodamineb_ = 98.7%	16
PI	GO	OSN	Rhodamine B	41.47 L m^−2^ h^−1^ MPa^−1^	*R*_Rhodamineb_ = 99.4%	131
PSf	Polyamide@NH_2__MIL-88B	RO	NaCl 2000 ppm	35.8 L m^−2^ h^−1^ 15.5 bar	*R*_NaCl_ = 92%	132
PSf	Sodium alginate (SA)	RO	NaCl 2000 ppm	27.8 L m^−2^ h^−1^ Mbar^−1^	*R*_NaCl_ = 99.2%	107
PAN	PEI/PPA	RO	NaCl 2000 ppm	20.7 L m^−2^ h^−1^ 0.6 MPa	*R*_NaCl_ = 98.7%	54
PSf	PPA	RO	Na_2_SO_4_ and NaCl 2000 ppm	2.86 L m^−2^ h^−1^ bar^−1^	*R*_NaCl_ = 97.3%	109
PSf	TpPa (COFs)	RO	NaCl 2000 ppm	16.78 L m^−2^ h^−1^ MPa^−1^	*R*_NaCl_ = 99.2%	70
Polyethylene (PE)	PDA/TA-Fe^3+^	FO	1 mol per L NaCl	71.2 L m^−2^ h^−1^	Js NaCl 0.03 g m^−2^ h^−1^	117
CTA	PDA/PEI	FO	1 mol per L NaCl	7.1 L m^−2^ h^−1^	Js NaCl 0.2 g L^−1^	48
PSf/TPU	UiO-66-NH_2_	FO	Cu^2+^ (500 μg L^−1^), tetracycline TC (CFU 10^3^–10^11^)	65 L m^−2^ h^−1^ bar^−1^	*R*_Cu^2+^_ = 99.53%, *R*_TC_ = 99.53%	133
PAN	Electrospun CNTs	FO	2 mol per L NaCl	49.2 L m^−2^ h^−1^	Js NaCl 7.2 g m^−2^ h^−1^	118
PVDF	TA–Fe^3+^	FO	2 mol per L NaCl	36.9 L m^−2^ h^−1^	Js NaCl 2.7 g m^−2^ h^−1^	50
Nylon	MXene	FO	1 mol per L LiCl	9.5 L m^−2^ h^−1^	Js LiCl 0.4 g m^−2^ h^−1^	119
Mixed cellulose ester	SWCNTs/PDA-PEI	FO	1 mol per L LiCl	51.3 L m^−2^ h^−1^	Js NaCl 7.9 g m^−2^ h^−1^	55
PSf	PDA/halloysite nanotubes	FO	1 mol per L NaCl	26.9 L m^−2^ h^−1^	Js NaCl 3.35 g m^−2^ h^−1^	134
Ceramic hollow fiber (CHF)	TiO_2_	Prevaporation	Dehydration	6.44 kg m^−2^ h^−1^	—	135
PAN	PEI/PPA	FO	0.5 mol per L NaCl	24.6 L m^−2^ h^−1^	Js NaCl 2.36 g m^−2^ h^−1^	105

Similar to the pressure filter loading method, the predeposition method can also produce wrinkles. For example, Gui *et al.* predeposited g-C_3_N_4_ nanofibers on the surfaces of PES membranes and obtained a high-quality PA layer with a folded structure using the ultrahigh hydrophilicity of the g-C_3_N_4_ nanofiber network intermediate layer. Due to the ultrahigh specific surface area of this special structure, the prepared NF membranes showed an ultrahigh water flux of 15.2 L m^−2^ h^−1^, a Na_2_SO_4_ retention efficiency of 98.9% (4 bar), and a significantly higher SO_4_^2−^/Cl^−^ selectivity.^108^

**Fig. 5:**
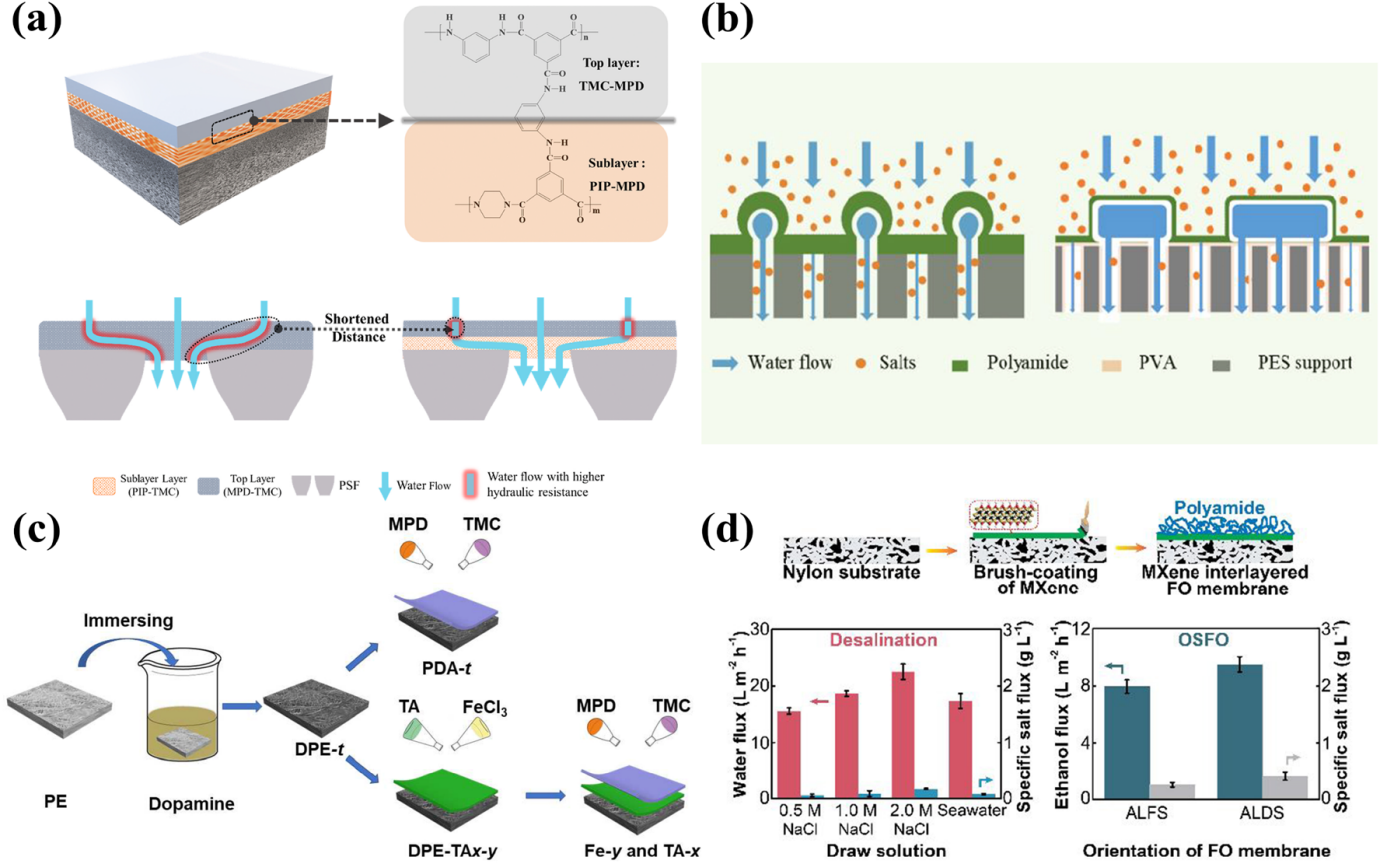
Predeposition of TFNi membranes with different nanomaterials. (a) The plain blue arrows represent water flow through a loose region (PPA). The blue arrows glowing red represent water flow through a dense region (PA) with higher hydraulic resistance [reprinted with permission from ref. 109. Copyright 2020, Elsevier]. (b) A high-performance NF membrane (TFC-P) was fabricated *via* IP on a PVA interlayered PES substrate [reprinted with permission from ref. 115. Copyright 2020, American Chemical Society]. (c) Schematic illustration of the modification of the PE substrate and the fabrication of PDA-t, Fe-y, and TA-x membranes [reprinted with permission from ref. 117. Copyright 2022, Elsevier]. (d) A high-performance MXene TFNi FO membrane was fabricated *via* a combination of a facile and scalable brush coating of MXene on nylon substrates and the IP process [reprinted with permission from ref. 119. Copyright 2020, American Chemical Society].

Since the PA layer formed by cross-linking the amine monomers with TMC in conventional TFC membranes is thick and has uneven pores, it is not conducive to the transport of water molecules. As shown in Fig. 5(a), Gan *et al.* solved this problem by introducing a sparser poly(piperazine amide) (PPA) interlayer between the PA layer and the PSf substrate. The obtained TFNi membrane showed a 2–2.5 times higher water flux than the TFC membrane while maintaining a higher retention rate.^109^ In addition, Yang *et al.* used PDA as an interlayer nanomaterial to investigate its effects on the selective permeabilities of TFNi membranes. The membrane separation performance was enhanced due to the combined effects of grooves formed by the PDA interlayer and indirect adhesion, with the grooves playing a more dominant role in increasing the roughness and cross-linking of the PA layer.^58^ The deposition time was substantially reduced.^110^ Yang *et al.* compensated for the oxidation of PDA in air and the long predeposition time by reacting PDA with PEI *via* a Michael addition reaction and used predeposition of a PDA/PEI sandwich on the PSf substrate to regulate the IP reaction of PIP and TMC and prepare TFNi membranes.^56^ Wang *et al.* similarly used PDA/PEI predeposition on a CTA substrate followed by IP to prepare TFNi membranes.^48^ However, the PDA predeposition process usually takes a long time. Therefore, Wang *et al.* studied the macrocyclic polyphenols of Noria and performed PEI predeposition on the surface of the substrate in only a few seconds.^111^ Positively charged TFNi membranes were prepared *via* the interaction of macrocyclic polyphenols of Noria and PEI.^106^

GO nanomaterials have received increasing attention in the field of membrane separation.^112^ Graphene quantum dots (GQDs) are zero-dimensional nanomaterials in the graphene oxide family.^113^ Liang *et al.* used GQDs and PEI predeposited on a PI substrate and subsequently obtained organic solvent nanofiltration membranes by performing IP reactions with low concentrations of aqueous and organic solutions.^16^

Polyvinyl alcohol (PVA) is a polymer with a linear molecular structure that can be used as an organic binder with good adhesion when dissolved in water.^114^ As shown in Fig. 5(b), Zhu *et al.* deposited PVA on the surface of a PES substrate, and a dense PA layer with a thickness of 9.6 nm formed on the PES-PVA surface due to its large specific surface area, good hydrophilicity, and high porosity. In addition, the TFNi membranes formed *via* PVA intercalation had smaller pore sizes and larger specific surface areas. Importantly, the PVA intercalation strategy was further advanced for possible application in NF membrane pilot lines by preparing membranes exhibiting stable water flux and high separation coefficients comparable to those of laboratory-scale TFNi membranes.^115^ Liang *et al.* used polyvinyl alcohol (PVA)-modified GO and subsequent glutaraldehyde postcrosslinking to control the IP reaction, which significantly reduced the separation layer thickness (to 15 nm) and roughness (to 5 nm). The authors obtained TFNi membranes with considerably increased chloride resistance.^116^

TA, a plant processing product, is susceptible to complexation reactions with metal ions and organic matter used to modify the surface of the substrate due to its abundant hydroxyl groups and hydrogen bonds. Zhang *et al.* prepared polyphenol interlayers using TA and DETA predeposition.^52^ As shown in Fig. 5(c), Xiao *et al.* introduced a TA–Fe^3+^ interlayer with a thickness of 7 μm on a PDA-modified polyethylene (PE) substrate and used the TA–Fe^3+^ interlayer to control the diffusion of MPD, leading to the formation of a unique worm-like morphology in the PA layer during the IP reaction. The substrate and the PA layer were closely connected by the TA–Fe^3+^ interlayer, which improved the stability of the TFNi membrane.^117^

Yao *et al.* formed PA layers by predepositing TA–Cu complexes on the surface of a PI substrate as interlayers to reduce the concentration of amine monomers in the organic solution during the IP reaction. The prepared TFNi membranes showed high permeability to organic solvents because of an activation process using DMF during fabrication.^49^ In addition, S. R. Razavi *et al.* prepared polyphenol interlayers using TA as a ligand on a PVDF substrate and crosslinking with Fe^3+^ ions. The effects of the TA–Fe^3+^ interlayer on the hydrophilicity and roughness of the membrane were investigated.^50^

Electrospun nanofibrous mats are widely used as substrates for forward osmosis membranes because they are unlikely to cause internal concentration polarization. Yang *et al.* prepared high-performance FO membranes by adding carbon nanotubes (CNTs) between a PA layer and an electrostatically spun polyacrylonitrile (PAN) substrate. The larger pore sizes of the substrate with the CNT interlayer provided a better platform for subsequent growth of the PA layer and maintained a stable osmotic pressure difference between the solutions on both sides of the membrane during operation.^118^

Since stable and large-scale production of two-dimensional nanosheets is difficult to achieve, Wu *et al.* developed a brush coating method similar to the spraying process used for large-scale preparation of MXene TFNi membranes. As shown in Fig. 5(d), the optimal permeate flux and retention rate of the FO membrane were investigated by controlling the number of brushing cycles used to place MXene on the nylon substrate and the concentration of the MXene solution. The as-prepared FO membrane showed a high water permeability of 31.8 L m^−2^ h^−1^ and a low specific salt flux of 0.27 g L^−1^ when using 2.0 mol L^−1^ sodium chloride as the draw solution. The obtained FO membranes were used for organic solvent recovery.^119^

**Fig. 6:**
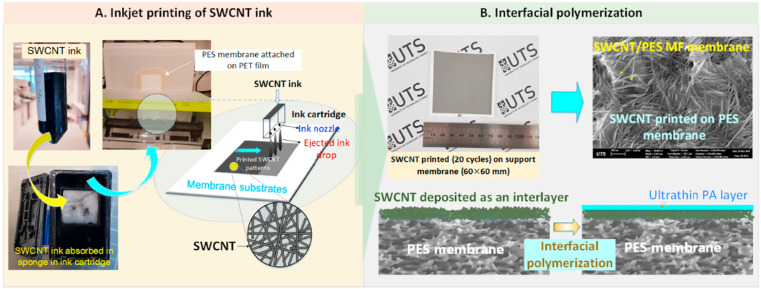
Schematic of (A) SWCNT predeposition on the PES membrane *via* inkjet printing and (B) deposition of an ultrathin PA layer onto the SWCNT-coated PES membrane *via* the IP reaction [reprinted with permission from ref. 19. Copyright 2021, Elsevier].

The inkjet printing process facilitates rapid deposition of inks with precise quantities and positions. The process may be automated with precise control of the spraying process, which facilitates mass production. As shown in Fig. 6, Wang *et al.* took advantage of the inkjet printing process and applied it to syntheses of nanofiltration membranes, in which single-walled carbon nanotubes (SWCNTs) were deposited by the inkjet printing process. The SWCNTs served as an interlayer between the PA layer and the PES substrate. The effect of the SWCNT interlayer thickness on the formation of the PA layer was investigated by controlling the number of times SWCNTs were printed on the PES substrate. Ultimately, the best membrane properties were obtained for an NF membrane synthesized by printing the SWCNTs 15 times.^19^

Future industrialization with the predeposition method is likely. On the one hand, most of the interlayer materials are metal compounds due to the use of the filtration loading method, which causes secondary leaching of metal ions in the subsequent water treatment process and secondary pollution of the environment. Second, if the separation layer of the NF membrane, PIPA, is used as the interlayer material and the PA separation layer of the RO membrane is subsequently formed on its surface, the preparation process may be expanded to enable rapid production. Therefore, industrial applications are more likely when producing TFNi membranes where the compound is predeposited on the surface of the substrate using methods such as spraying or brushing.

### 4.3 *In situ* growth

The shortcoming resulting from loading a PA layer onto the surface of the substrate by filtration is weak interfacial bonding between the substrate and the PA layer, which may lead to detachment of the PA layer and leaching of the nanomaterials during long-term water treatment.^136^ Nanomaterials were grown directly on the surface of the substrate during synthesis to solve this problem.

The choice of material and substrate is considered. *In situ* growth is usually performed directly on the surface of the material, using the substrate as a support during the synthesis process. Most of the materials are synthesized at high temperatures and under acidic conditions. Therefore, the choice of substrates and nanomaterials is limited considering the mechanical strength, acid resistance and temperature tolerance range of the substrate.

For example, Song *et al.* grew TiO_2_ on the surface of a PSf substrate *via* an *in situ* growth method. Since the synthesis process requires the substrates to be immersed in the organic solvent ethanol for a long time, good resistance to organic solvents is needed. The TiO_2_ interlayer prepared using the *in situ* growth method significantly improved the surface hydrophilicity of the PSf substrate and promoted aggregation of the amine monomers in the aqueous solution. TiO_2_ also slowed the polymerization of amine monomers in the organic phase with the TMC interface and promoted the formation of ultrathin PA layers (13 nm).^137^

**Fig. 7:**
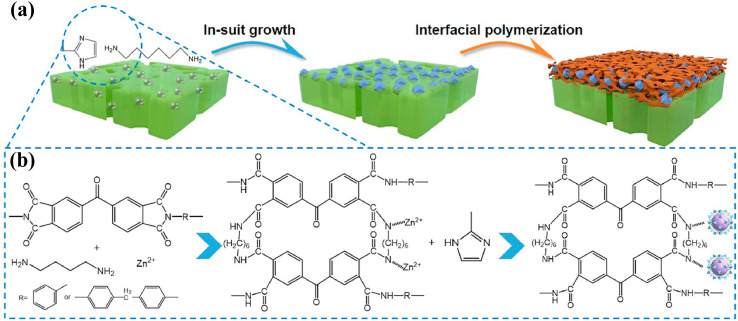
(a) Schematic illustrating the fabrication of TFNi membranes featuring an interlayer of ZIF-8 nanocrystals and (b) mechanism, including the crosslinking reaction and nucleation of the ZIF-8 nanocrystals [reprinted with permission from ref. 60. Copyright 2021, Elsevier].

MOF porous nanomaterials have attracted extensive research interest in recent years.^138,139^ The current methods used to prepare MOF interlayers are coating,^140^ filtration^141^ and layer-by-layer self-assembly.^142^ However, the disadvantage of these preparation methods is weak interfacial compatibility due to poor adhesion between MOFs and the substrate. Crosslinking with the polymer is enhanced by heating the PI substrate, which does not affect the stability and flux of the membrane.^143^ As shown in Fig. 7, Wei *et al.* cross-linked PI with hexamethylene diamine and grew ZIF-8 nanomaterials *in situ* on its surface, which effectively prevented agglomeration of ZIF-8 on the PI surface.^60^ In addition, Chen *et al.* synthesized HKUST-1 on the PI surface using a similar *in situ* growth method. The hydrophilicity and porosity of the substrate were improved by intercalating HKUST-1 nanomaterials, and the obtained TFNi membrane was subsequently used for the recovery of bright blue g250 dye from organic solvents.^61^

Methods for the chemical modification of substrates, such as nylon, PSf, PAN and materials grown on their surfaces, are also emerging. For example, Hu *et al.* grafted a PAP-like molecule onto the surface of a substrate with a diazonium-induced anchoring process (DIAP).^144^ The PAP layer significantly increased the porosity and hydrophilicity of the substrate surface but had little effect on its surface pore size structure. The effects of PAP layers on the distribution, release and uptake of aqueous phase piperazine (PIP) monomers during the IP reaction were also systematically investigated. He *et al.* used nylon membranes as the substrate, which were first hydrolyzed to carry free amino groups on their surfaces and then reacted them with TMC to obtain negatively charged nylon membranes. Subsequently, TFNi membranes were prepared using the *in situ* growth of sulfonated COF intercalated nanomaterials.^66^ Chen *et al.* enhanced the surface cross-linking of PEI with the carboxylated PAN substrate, and the PEI intercalation layer improved the hydrophilicity of the substrate. This approach enabled the substrate to adsorb more amine monomers from aqueous solution and control the IP reaction with the release rate.^53^

TpPa is a COF, and it is often used in membrane separations because it can be synthesized at normal temperature and pressure and loaded onto the surface of the substrate through *in situ* growth without affecting the membrane structure.^66–71^ Wang *et al.* constructed structurally complete and stable TpPa interlayers on a PI substrate in a manner similar to that reported by Yang *et al.* to control the IP process. The COF interlayer was grown *in situ* using a low concentration of precursor solution, and the PA separation layer was prepared using low-concentration aqueous and organic solutions. The obtained membranes were used for organic solvent nanofiltration due to the inherently high stability of COFs toward organic solvents and the low roughness of the obtained PA layer.^67,145^

**Fig. 8:**
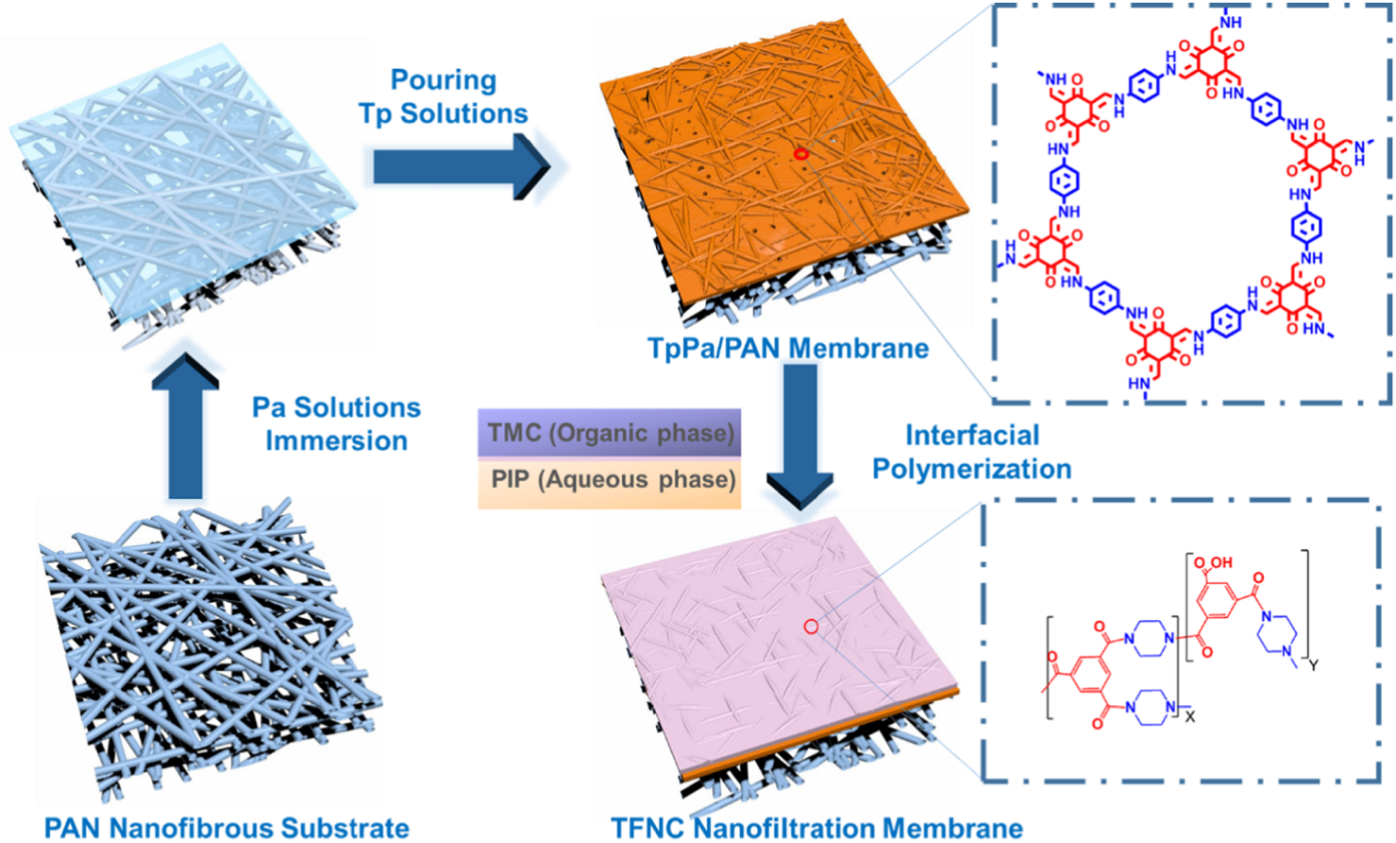
Schematic diagram illustrating the method for preparing the PA/TpPa/PAN TFNC membrane [reprinted with permission from ref. 69. Copyright 2021, Elsevier].

As shown in Fig. 8, Zhang *et al.* introduced TpPa on the surface of a PAN substrate and performed an IP reaction on its surface to prepare a TFNi membrane with a thin PA layer.^69^ Li *et al.* assembled COF interlayers on the surfaces of a PSf substrate and then generated a PA layer with the IP reaction, and the prepared TFNi membranes were used in a reverse osmosis process.^70^ In addition to the flat PSf substrate, Jiang *et al.* grew COFs *in situ* on the surface of a PSf HF substrate and constructed HF membranes exhibiting significantly improved separation performance.^68^ Overall, COF intercalation modulates the pore structure of the substrate and controls the uptake and release of amine monomers during the IP reaction, resulting in a thinner and more highly cross-linked PA separation layer.

TFNi membranes were prepared using the electrospray IP (EIP) method, which enhanced the separation performance by increasing the crosslinking of PA layers through electrospraying aqueous and organic solutions.^146,147^ Yang *et al.* prepared Span 80 intercalated TFNi membranes by electrospraying and improved both the water treatment stability and separation performance of the TFNi membranes.^148^ Combining electrospraying and sandwiching methods represents a useful approach to prepare high-performance TFNi membranes.

The aforementioned *in situ* growth method has fewer options available due to the high requirements for substrate and interlayer materials. As shown in Table 3, fewer studies have used this approach compared to pressure filtration and predeposition. In addition, the *in situ* growth method has a lower material utilization rate during the TFNi membrane preparation process. The process is more complicated, resulting in a lower experimental reproducibility. Therefore, its industrialization potential is relatively limited.

**Table 3:** Summary of *in situ* growth methods for preparing TFNi membranes

Substrate material	Interlayer nanomaterials	Membrane type	Operating conditions (applied pressure and feed solution)	Permeability (L m^−2^ h^−1^ bar^−1^)	Rejection (%)	Reference
Nylon	SCOF	NF	Na_2_SO_4_ 1000 ppm	36.6 L m^−2^ h^−1^ bar^−1^	*R*_Na_2_SO_4__ = 99.6%	149
PSf	SiO_2_	NF	Na_2_SO_4_ 1000 ppm	14.5 L m^−2^ h^−1^ bar^−1^	*R*_Na_2_SO_4__ = 98.7%	137
PI	HKUST-1	NF	Brilliant blue G 250 (858.05 g mol^−1^)	9.59 L m^−2^ h^−1^ bar^−1^	*R*_dyes_ = 98.8%	61
PES/SPSf	Chitosan nanoparticles (CSPs)	NF	NaCl, MgSO_4_, Na_2_SO_4_ and MgCl_2_ 1000 ppm	45.2 L m^−2^ h^−1^ bar^−1^	*R*_Na_2_SO_4__ = 99.3%	150
PSf	PDA/PEI or TA/PEI or ZIF-8/PEI	NF	NaCl, MgSO_4_, Na_2_SO_4_ and MgCl_2_ 1000 ppm	141.238 L m^−2^ h^−1^ bar^−1^	*R*_Na_2_SO_4__ = 98.4%	151
CA (cellulose acetate)	PEI-MWCNT	NF	MgCl_2_ (Mg^2+^), Co(NO_3_)_2_ (Co^2+^), Zn(NO_3_)_2_ (Zn^2+^), Pb(NO_3_)_2_ (Pb^2+^), CuCl_2_ (Cu^2+^), Ni(NO_3_)_2_ (Ni^2+^), and CdCl_2_ (Cd^2+^)1000 ppm	16.5 L m^−2^ h^−1^ bar^−1^	*R*_MgCl_2__ = 95% *R*_Co(NO_3_)_2__ = 80%, *R*_Zn(NO_3_)_2__ = 73% *R*_Ni(NO_3_)_2__, CuCl_2_, Pb(NO_3_)_2_ < 20%	152
PSf	ZIF-8	NF	Congo red	27.1 L m^−2^ h^−1^ bar^−1^	*R*_dyes_ = 99.8%	21
PSf	PDA/UiO-66-NH_2_	NF	NaCl, MgSO_4_, Na_2_SO_4_ and MgCl_2_ 1000 ppm	12.2 L m^−2^ h^−1^ bar^−1^	*R*_Na_2_SO_4__ = 98.1%	57
PSf	PEI/TA	NF	Na_2_SO_4_ 1000 ppm	65 L m^−2^ h^−1^ bar^−1^	*R*_Na_2_SO_4__ = 99%	51
PSf	PAP	NF	MgSO_4_, Na_2_SO_4_ and MgCl_2_ 2000 ppm	6.5 L m^−2^ h^−1^ bar^−1^	*R* = 98%	144
SPEEK	GO/PEI/GA	OSN	AF (585.54 g mol^−1^), CR (696.68 g mol^−1^), MB (799.8 g mol^−1^), RB (1017 g mol^−1^)	Water 30 L m^−2^ h^−1^ bar^−1^, acetone 27.16 L m^−2^ h^−1^ bar^−1^, methanol 13.88 L m^−2^ h^−1^ bar^−1^, ethanol 9.94 L m^−2^ h^−1^ bar^−1^, IPA 7.42 L m^−2^ h^−1^ bar^−1^	*R*_dyes_ = 99%	12
PSf	Fe(BTC)	RO	NaCl	2.93 L m^−2^ h^−1^ bar^−1^	*R*_NaCl_ = 96.8%	153
PI	ZIF-8	OSFO	Ethanol	Ethanol 2.10 L m^−2^ h^−1^	*R*_polystyrene_ = 89.8%	60
PAN	PEI	FO	MgCl_2_ 500 ppm	16.1 L m^−2^ h^−1^ bar^−1^	Js NaCl 1.25 g m^−2^ h^−1^	53
Nylon	SCOF	FO	1 mol per L NaCl	26.7 L m^−2^ h^−1^	Js NaCl 1.08 g m^−2^ h^−1^	66

## 5. TFNi membrane characterization technology

The properties of TFN membranes are determined by the polyamide layer on the support surface and therefore require qualitative analyses, quantitative analyses and surface observations. Several characterization methods are presented below to determine the thickness of the polyamide layer formed on the membrane surface, its denseness, and the effect of the interlayer material interfacial polymerization on the IP process.

### 5.1 Spectroscopic techniques

Spectroscopy is a general term for a class of characterization techniques that use electromagnetic radiation to obtain relevant information. The process of PA layer formation in TFNi membranes has been analyzed with various spectroscopic techniques.

Among them, Fourier transform infrared (FT-IR) spectroscopy relies on the detection of molecular bond vibrations to determine the functional groups of interest but differs because it provides more timely feedback on the detection results than conventional infrared spectroscopy. *In situ* FT-IR spectroscopy is an effective tool for studying the IP reactions between solid and liquid phases^154^ that provides information on the mechanism and kinetics of IP reactions.^155^

For example, Ren *et al.* and Han *et al.* used a homemade FT-IR cuvette to monitor the IP reactions of alicyclic methacrylate hydrogels used to control drug release.^156,157^ In addition, the thickness of the PA layer was detected in real time by fitting the absorption intensities of characteristic bands in the FT-IR spectrum with a mathematical equation relating them to the thickness of the PA layer.^158^

**Fig. 9:**
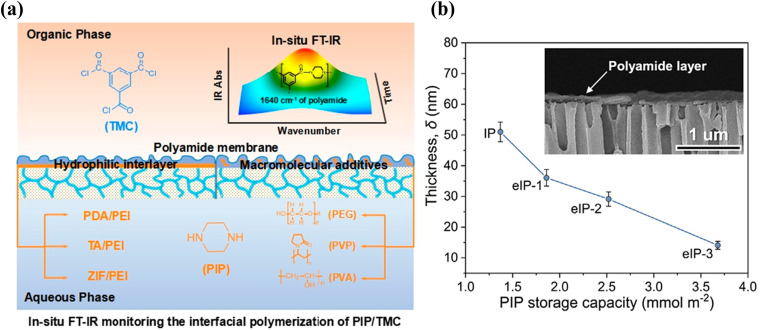
(a) *In situ* FT-IR spectroscopic monitoring of the IP reaction of PIP and TMC for the introduction of hydrophilic interlayers on the pristine PSf substrate or macromolecular additives in the aqueous solution composed of PIP [Reprinted with permission from ref. 151. Copyright 2020, Molecular Diversity Preservation International]. (b) Thicknesses of PA membranes prepared using conventional IP and eIP. (Inset) Cross-sectional morphology of the ultrathin polyamide membrane on the AAO substrate [reprinted with permission from ref. 162. Copyright 2021, Elsevier].

As shown in Fig. 9(a), Yang *et al.* used *in situ* infrared spectroscopy to study the IP reaction in real time and successfully established a relationship between the thickness of the PA layer and the rate of the IP reaction.^151^ IP reactions on the PSf substrate were monitored using FT-IR spectroscopy. Usually, FT-IR spectroscopy can monitor a reaction for a few hours. However, because of the short duration of the IP reaction, the monitoring time is usually fixed between 0–300 s with a minimum interval of 15 s. The response of the IP reaction rate to the addition of nanomaterials or polymer additives to the surface of the substrate was observed.

The effect of nanomaterial intercalation on the rate of amine monomer adsorption and diffusion in aqueous solution was determined.

Song *et al.* studied the diffusion of PIP from the aqueous phase to the organic phase with UV–vis spectroscopy. Specifically, the aqueous solution containing dispersed SiO_2_ was poured into a beaker, and the organic solution was added slowly. After 30 s of the reaction, 2 mL of the organic solution were poured into a quartz cuvette. The concentration of amine monomer in hexane (diffusion rate) was determined by observing the intensity of the absorption peak for PIP at 212.5 nm.^159–161^

The concentration of PIP in the organic phase was determined with UV–vis absorption spectrometry, as shown in Fig. 9(b), and the amount of PIP adsorbed by the intercalated nanomaterials was determined based on the diffusion kinetics of PIP. The effects of different substrates on the diffusion rate (*D*_r_) of PIP were calculated using eqn (1):^162^1
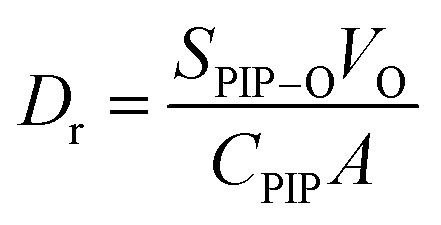
where *S*_PIP–O_ (g L^−1^ min^−1^) represents the rate of diffusion of PIP from the membrane surface into the organic solution, *V*_O_ represents the volume of the organic solution (mL), *C*_PIP_ represents the capacity of the substrate to store PIP (g m^−2^), and A represents the surface area of the substrate (m^2^).

The kinetic model proposed by Freger for PA layer formation by IP was used to evaluate the PA layer thickness. At the same diffusion time, the thickness of the PA layer is inversely proportional to the amine monomer concentration at reaction equilibrium and proportional to the one-third power of the diffusion coefficient for transfer of the amine monomer into the organic phase. The Freger kinetic model for the formation of the separation layer has been used to calculate the thickness of the separation layer with eqn (2):^163^2
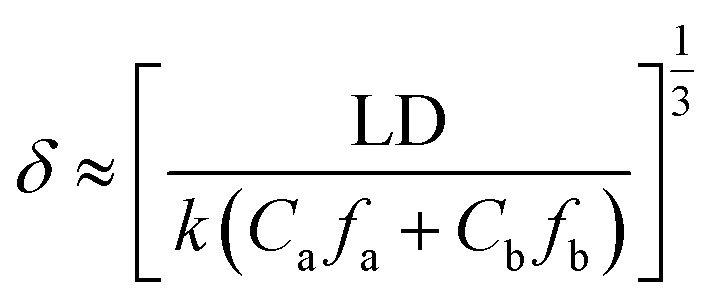
where *L* (m) is the thickness of the interfacial diffusion boundary layer; *D* (m^2^ s^−1^) is the diffusion rate of the amine monomer; *k* (L mol^−1^ s^−1^) is the rate constant for the reaction between the two monomers; *C*_a–b_ (mol L^−1^) and *f*_a–b_ (mol L^−1^) are the equilibrium concentrations of the amine and acyl chloride monomers, respectively; and *f*_a_ and *f*_b_ are the equilibrium functional groups of the amine and acyl chloride monomers, respectively.

The experimental and simulation results showed that when the IP reaction was performed on the surface of the substrate with a high loading of the nanomaterial interlayers, the rate for diffusion of the amine monomers from the aqueous phase to the organic phase decreased; accordingly, a thinner and more cross-linked PA layer was formed.^137^

### 5.2 X-ray spectroscopy

**Fig. 10:**
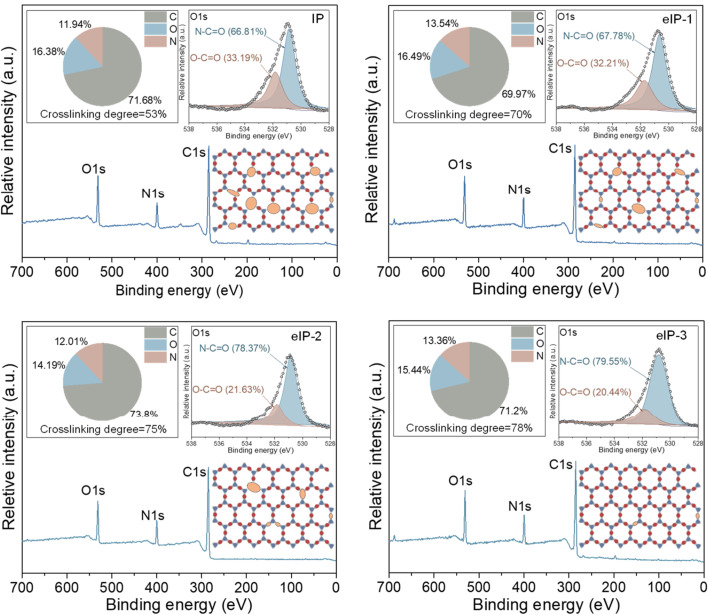
Elemental compositions (top left panel), high-resolution O 1s core spectrum (top right panel) and crosslinking structure (bottom right panel) of polyamide membranes [reprinted with permission from ref. 162. Copyright 2021, Elsevier].

X-ray photoelectron spectroscopy (XPS) uses electromagnetic radiation to quantify elemental compositions. XPS characterization plays an important role in studying TFNi membranes by analyzing the elemental composition to determine whether the nanomaterial was successfully intercalated. In addition, the elemental composition and chemical bonding state of the membrane surface can also be analyzed. As shown in Fig. 10, You *et al.* enhanced crosslinking of the PA layer by electrically driving the IP reaction, which increased the N–C=O content from 66.8% to 79.6% and decreased the O–C=O content from 33.2% to 20.4%.^162^

The crosslinking degree of the TFNi membrane was calculated using XPS to compare the elemental concentrations of the different membranes with eqn (3):^164^3
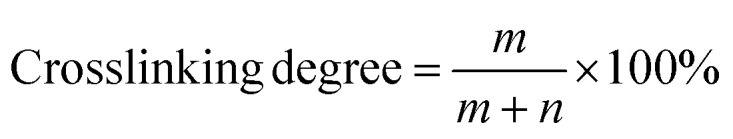
where *m* denotes the cross-linked structure and *n* is the linear structure of the polyamide, which was calculated using eqn (4):4
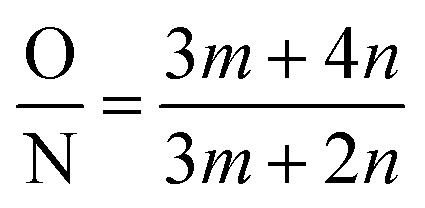
where O and N represent the atomic percentages of oxygen and nitrogen, respectively, as measured using XPS.

Microscopy is a technique enabling direct visualization and determination of the physical characteristics of a PA layer on a membrane surface. The different microscopy techniques, including atomic force microscopy (AFM), transmission electron microscopy (TEM) and scanning electron microscopy (SEM), are discussed below.

AFM, which is also referred to as scanning probe microscopy (SPM), uses physical interactions of the probe tip with the sample surface to obtain the corresponding image. This method does not require a vacuum, can be used in both gaseous and liquid environments, and does not require electrically charged or metal-coated samples. As a result, the measurements are convenient and less destructive to the sample. In addition, AFM provides additional advantages for analyzing the surfaces and microstructures of objects and quantifying the thicknesses of PA layers.

**Fig. 11:**
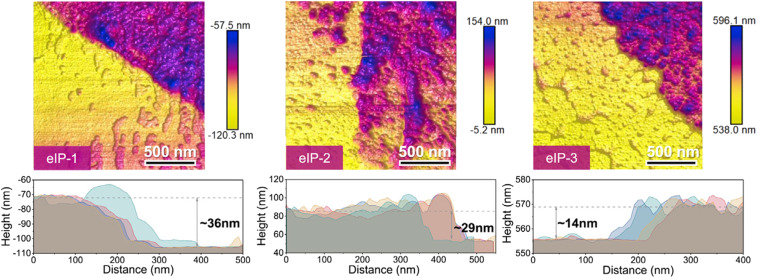
AFM images and corresponding height profiles for substrate-free eIP polyamide membranes [reprinted with permission from ref. 162. Copyright 2021, Elsevier].

You *et al.* immersed a TFN membrane in DMF, dissolved the substrate with an organic solvent, and then washed it with ethanol to obtain a PA layer without substrate to characterize the thickness of the PA layer. This material was transferred to an anodic aluminum oxide (AAO) substrate to observe the complete structure of the PA layer, which enabled the measurement of the membrane thickness using AFM. As shown in Fig. 11, the thicknesses of eIP-1, eIP-2, and eIP-3 were ∼36 nm, ∼29 nm, and ∼14 nm, respectively, according to the height distributions of the AFM images.^162^

**Fig. 12:**
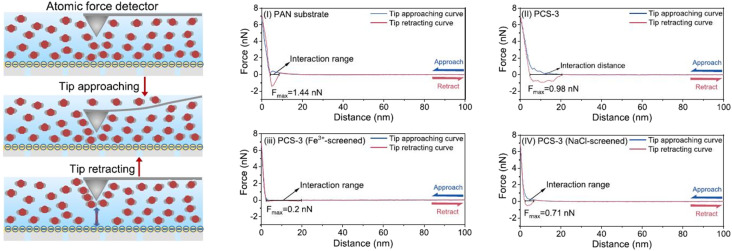
Schematic diagrams showing measurements of the interaction between PIP and the substrate with an atomic force detector and force–distance curves for wetting of substrate surfaces. The atom-scale PIP-monomer interactions with PAN(i), PCS-3 (ii), PCS-3 (Fe^3+^-screened) (iii), and PCS-3 (NaCl-screened) (iv) were measured [reprinted with permission from ref. 162. Copyright 2021, Elsevier].

In addition to measuring the thickness of the PA layer, the magnitude of the interaction force between the amine monomer and the surface of the substrate can also be measured with a solution test. Shen *et al.* performed AFM in probe tapping mode.^165^ As shown in Fig. 12, the amine monomer was adsorbed by the probe tip when the AFM probe was immersed in the aqueous solution. When the measurement started, the amine monomer adsorbed on the probe tip established an interaction force with the substrate. Subsequently, the probe tip with the adsorbed amine monomer was retracted from the surface of the substrate to determine the resistance of the interaction forces established between the amine monomer and the substrate. This amine monomer substrate interaction force can be characterized quantitatively at the atomic scale by constructing a force–distance curve.

### 5.3 SEM and TEM

SEM provides an image of the surface topography of the test object by focusing an electron beam on the sample surface and detecting secondary electrons, backscattered electrons and X-rays emitted by the sample.^166^ SEM has the advantage of shorter image acquisition and capture time than AFM. In addition, sample preparation for SEM is easier than for TEM. Therefore, SEM is widely used by researchers.

Song *et al.* used SEM to observe the nodular structure of a TFC membrane surface with closely dispersed granular bumps. In addition, the morphological characteristics of the SiO_2_ TFNi membrane were observed, and the surface microstructure was noticeable and contained abundant ridge-like nanoribbons. With increases in the amount of nanomaterial added, the surface of the substrate in the TFNi membrane showed more structural features of a nanoribbon PA layer.^137^

TEM provides images with a resolution close to the atomic scale and provides two different types of images: dark field and bright field. Dark-field images are generated by diffracted electrons, and thus only the details of the crystal structure are reflected. In bright-field images, beam attenuation at different sample densities is caused by transmitted electrons. TEM techniques have been used to characterize the surface properties of films. The specific distribution of nanomaterials in a PA layer have been observed using TEM. For example, Yang *et al.*^18^ used TEM to show that hydrophilic AgNP nanomaterials attract surrounding aqueous phase amine monomers and subsequently block the formation of IP reactions around them to reduce the thickness of the PA layer and increase cross-linking.

### 5.4 Quartz crystal microscale dissipation (QCMD)

The QCMD technique has been widely used to study the thicknesses of materials deposited on solid surfaces.^167^ Song *et al.*^168^ investigated the deposition rates of ultrathin PA layers measuring 8, 15, and 25 nm with the QCMD technique. The authors first washed the SiO_2_ chip sensor with deionized water and then soaked the sensor in an aqueous solution. An organic solution was then applied to the sensor surface. The amount of PA deposited was calculating by recording the adsorbed mass Δ*m*_s_ (ng cm^−2^) on the sensor surface, and Δ*m*_s_ was determined using eqn (5):^169^5
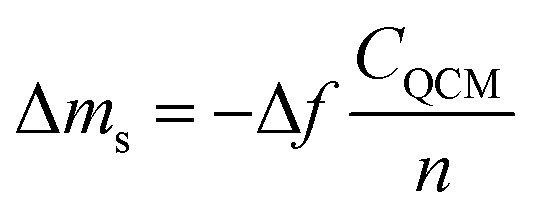

6 Thickness = Δ*m*_s_/*ρ*

where Δ*f* denotes the adsorption frequency, *C*_QCM_ (=17.7 ng cm^−2^ Hz^−1^, *f* = 5 MHz) is the mass constant, and *n* (=1, 3…) is the overtone constant. By assuming that the density of the PA layer is *ρ* (=1 g cm^−3^) independent of the aqueous phase concentration, the thickness of the PA layer (thickness) can be estimated using eqn (6).

**Fig. 13:**
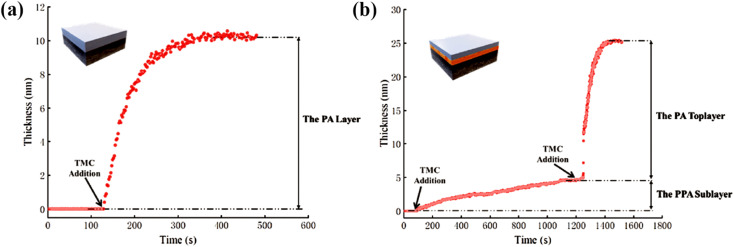
QCMD characterization of the evolution process of selected layers in the (a) uPA layer and (b) A-uPA sublayer and a top PA layer [reprinted with permission from ref. 109. Copyright 2020, Elsevier].

In addition, as shown in Fig. 13, Gan *et al.* used QCMD to monitor changes in quality during the formation of PA layers.^109^ The process was divided into two stages. In the first stage, a PPA sublayer was generated *via* the reaction of an aqueous solution of PIP (0.05 wt%) with TMC/hexane (0.01 wt%). In the second stage, the MPD aqueous solution (0.1 wt%) reacted with the TMC/hexane (0.02 wt%) solution directly on top of the sublayer to form the PA layer. The change in sensor mass due to the formation of the PA layer was quantified by analyzing the frequency change with the Q-Tool software using the Sauerbrey equation.

Surface wettability depends on the interfacial tension of three interfaces, solid–liquid, solid–gas and liquid–gas, which reflects the interactions between liquid and solid phase surfaces. During the operation of the membrane, the contact angle of the membrane is closely related to the permeability, anti-pollution and other properties of the membrane and is the main index used to judge the hydrophobicity of the membrane (when the contact angle >90°, the membrane surface is superhydrophobic). The main method for measuring the contact angle is the static drop method, which is widely used because it is quick and easy to apply.

## 6. Conclusions and outlook

**Fig. 14:**
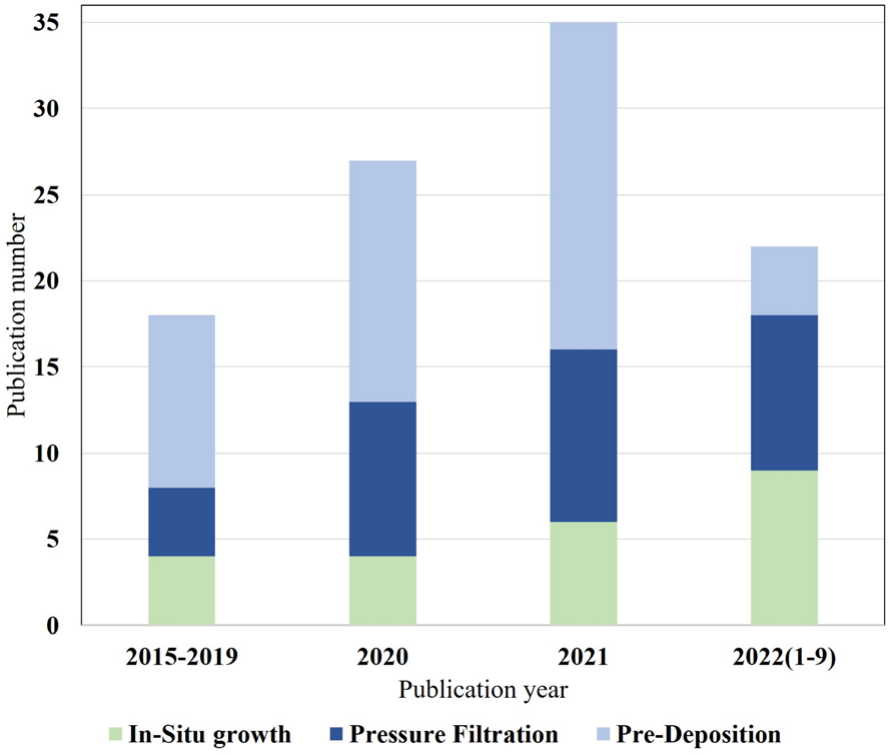
Statistical data on *in situ* growth, pressure filtration and predeposition methods are based on the ratio of different preparation methods to total publications.

This review presents three methods for the preparation of TFNi membranes, namely, pressure filtration, deposition and *in situ* growth, as well as several common methods for characterizing TFNi membranes. Each preparation method has its own advantages and disadvantages. As shown in Fig. 14, the number of articles using different methods to fabricate TFNi membranes that were published from 2015–2022 is summarized, and the total number of papers is increasing annually.

The proportion of TFNi membranes prepared using pressure filtration loading methods has likewise increased. This phenomenon may be attributed to the fact that the pressure filtration process is more controllable than the other two methods, and the variety of substrate and interlayer materials available makes it more appropriate for laboratory-scale studies. However, the pressure filtration method of loading the interlayer material with a limited effective filtration area and the cost prevent its production from being scaled for industrial applications.

For the predeposition method, a broader selection of intercalation materials is available, including some polymers such as PDA or PPA. The advantage is that the effective filtration area of TFNi membranes prepared using predeposition can be quickly scaled up compared to pressure filtration. The preparation process is equally convenient to the industry as it is controlled.

The *in situ* growth method is the technique that has received the least attention from researchers. This phenomenon may be attributed to the choice of materials and substrates. *In situ* growth is generally conducted directly on the material surface using the substrate as a support during synthesis. Most materials are synthesized under high-temperature and acidic conditions. Therefore, the selection of substrates and nanomaterials is limited. Moreover, TFNi membranes prepared using both *in situ* growth and pressure filtration methods are subject to the leaching of materials during water treatment, causing secondary environmental contamination.

Based on the summary provided above, we will discuss several potential considerations for future research on TFNi membranes and their development trends.

Regarding the selection of materials, we should consider the leaching of nanomaterials and cost, regardless of the method used to prepare TFNi membranes. Therefore, we should opt for environmentally friendly and low-cost intercalation materials. Since TFNi membranes are more suitable for RO/NF due to their smaller structural parameters and denser polyamide layer than TFN membranes and commercialized TFN membranes are already on the market,^170,171^ the commercialization of TFNi membranes is very promising.

Several potential methods for the industrial preparation of TFNi membranes are worth noting. By sacrificing the intercalation material, the modified polyamide layer is maintained, and the concern of secondary leaching in the water treatment process is avoided. Furthermore, when using the PPA layer of the NF membrane as the interlayer material, we are able to reduce the structural parameters and further improve the RO membrane performance. In addition, this method can be quickly scaled up for manufacturing. In addition, the spraying of the interlayer material with mechanical equipment enables the precise control and rapid scale up of production.

## Conflicts of interest

The authors declare that they have no known competing financial interests or personal relationships that could have appeared to influence the work reported in this paper.

## Supplementary Material
